# Essay on the Mechanism and Management of Parturition, in the Shoulder Presentation

**Published:** 1853

**Authors:** 


Art. XII.—*Essay on the Mechanism and Management of Parturition, in the Shoulder Presentation.* By William M. Boling, M. D., of Montgomery, Ala.

Dr. Boling is one of the most industrious physicians in our country. He is at once a writer and a hard-working practitioner. His papers are always acceptable to the profession, for they are the result of reading, reflection, and experience. The monograph before us is the fruit of much research and observation. All the authorities have been consulted, and every expedient proposed in the accident under consideration, is weighed and criticized. It is a full and thorough presentation of the subject, and will be read with instruction by those who are not already versed in such obstetrical questions. Dr. Boling proves by numerous cases that even in these unfortunate presentations, delivery may take place without the assistance of the accoucheur:—

"Besides the eight cases mentioned, as occurring under my own observation, in one of which the patient was delivered by the efforts of nature, and in three at least of which delivery in the same manner, there is good reason to believe, might have ultimately taken place, I have been furnished by my professional acquaintances with the particulars of two other cases, being the only ones of which I have heard among them. One of these was delivered after repeated efforts to turn, by two gentlemen in attendance; and while they had stepped aside to discuss the further treatment of the case, by the natural efforts, aided by traction on the protruding arm, made by a negro midwife, who had had the management of the case before the physicians were called, and who boasted not a little of the success of her practice, after the doctors, as she insisted, had "given the patient out." The other case was also delivered by the natural efforts, aided by traction on the arm, made by the husband himself, of the patient, during the absence of the physician, who had left for the purpose of procuring a pair of forceps with which to effect delivery. Thus, three out of ten cases at least, may be regarded as having been delivered by the natural powers."

